# The 2020 coronavirus lockdown and seismic monitoring of anthropic activities in Northern Italy

**DOI:** 10.1038/s41598-020-66368-0

**Published:** 2020-06-10

**Authors:** Piero Poli, Jacopo Boaga, Irene Molinari, Valeria Cascone, Lapo Boschi

**Affiliations:** 1 ISTERRE, Université de Grenoble, Grenoble, France; 2 Dipartimento di Geoscienze, Università degli Studi di Padova, Padova, Italy; 3 Istituto Nazionale di Geofisica e Vulcanologia, via Donato Creti 12, Bologna, Italy; 4 Sorbonne Université, CNRS, INSU, Institut des Sciences de la Terre de Paris, ISTeP UMR 7193, F-75005 Paris, France

**Keywords:** Geophysics, Seismology

## Abstract

In March/April 2020 the Italian government drastically reduced vehicle traffic and interrupted all non-essential industrial activities over the entire national territory. Italy thus became the first country in the world, with the exception of Hubei, to enact lockdown measures as a consequence of the COVID-19 outbreak and the need to contain it. Italy is also a seismically active area, and as such is monitored by a dense permanent network of seismic stations. We analyse continuous seismic data from many stations in northern and central Italy, and quantify the impact of the lockdown on seismic ambient noise, as a function of time and location. We find that the lockdown reduces ambient noise significantly in the 1–10 Hz frequency range; because natural sources of seismic noise are not affected by the lockdown, the seismic signature of anthropic noise can be characterised with unprecedented clarity, by simply comparing the signal recorded before and after the lockdown. Our results correlate well with independent evaluations of the impact of the lockdown (e.g., cell phone displacements), and we submit that ambient-noise seismology is a useful tool to monitor containment measures such as the coronavirus lockdowns.

## Introduction

On March 9, 2020, the Italian government issued a decree prohibiting movement in public places except for justifiable work reasons, basic necessities and health emergencies, canceling sporting events and public gatherings, closing schools, universities, and recreational facilities on the entire national territory. This followed smaller-scale lockdowns of eleven municipalities in the North (February 21), soon expanded to the entire region of Lombardy and fourteen neighbouring provinces (March 8), decided in an attempt to contain a major outbreak of the COVID-19 pandemic.

On March 22, through another decree, all non-essential industries were closed down throughout the country; inter-city movement was further restricted, requiring travellers to provide justification and documentation to authorities on any movement between cities. At the time of completing this manuscript, all these lockdown measures are still ongoing.

It has been pointed out that containment measures in Belgium (for example) have resulted in a conspicuous drop in the continuous signal recorded by broadband seismic stations^1^. This means that all the anthropic activities interrupted by the lockdown contribute importantly to “the hum of vibrations in the planet’s crust;” it also means, then, that the lockdown is an opportunity for students of “seismic ambient noise”^2,3^ to clearly separate its anthropic vs. natural components, normally intermingled and not easily distinguishable.

Ambient noise, or seismic signal recorded in the absence of earthquakes, is known to largely consist of “microseisms” resulting from the coupling between oceans and the solid earth at frequencies mostly below 1 Hz^4^. At relatively high frequencies, however, it also includes the so-called “anthropic” or “cultural” noise^5–7^ associated with human activities at or near the surface of the Earth, namely machinery in power plants and factories, and train and road traffic. Anthropic noise is thought to include frequencies from 1 to 10 Hz approximately, attenuating quickly with distance (a few km) and disappearing quickly with increasing depth; its total energy changes daily (day vs. night), weekly (working days vs. weekends), and with the occurrence of holidays (the Christmas break is particularly prominent in many countries). Just like the “natural”, low-frequency noise, that provides unique observations of surface-wave propagation allowing to map the structure of the Earth’s lithosphere^8^, anthropic noise is of interest to geoscientists, as it can be used at relatively small scales, for instance in mapping and monitoring efforts^9,10^.

**Figure 1:**
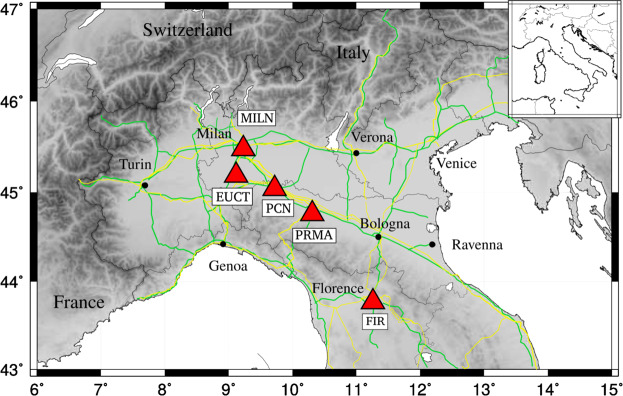
All data analysed in our study were recorded at the broadband seismic stations marked here by red triangles and acronyms; all stations are part of the Italian National Seismic Network operated by the Istituto Nazionale di Geofisica e Vulcanologia. Green and yellow lines denote major highways and railways, respectively.

While earlier studies have attempted to characterise high-frequency seismic noise^7,11–14^, the current lockdown of industrial activities and reduction in road and train traffic in Italy is an unprecedented opportunity to discriminate it from ambient noise of natural origin. Italy is a highly industrialized and urbanized country, densely covered with non-stationary noise sources^15^, such as traffic and industry-induced vibration^16^. This is particularly true in its northern regions, which account for 70% of the country’s entire industrial output, and where lockdown measures have been enacted earlier than everywhere else in Europe. We analyse continuous data from an array of broadband seismic stations, located in the vicinity of known industrial districts in Lombardy, Emilia-Romagna and Tuscany (Fig. 1); we identify the spectral signature of the March 2020 lockdown, and take advantage of the lockdown to quantify and evaluate the spectral signature of anthropic activities. Importantly, measuring the overall reduction in seismic energy associated with the lockdown is also a way to quantify its effects; this is relevant to governmental entities, wishing to monitor the effectiveness of the measures being taken.

## Data

We downloaded publicly available, continuous, three-component seismic recordings from a set of permanent broadband stations, part of the Italian National Seismic Network operated by the Istituto Nazionale di Geofisica e Vulcanologia^17^. All instruments have a flat response at frequencies between ~0.01 and ~10 Hz, or broader; we remove (“deconvolve”) instrument response from the data prior to our analysis. The locations of stations employed in most of our study are shown in Fig. 1. Stations were selected based on their proximity to industrial districts; in particular, MILN is located near the city of Milano, with a particularly high concentration of vehicle traffic and industrial activities.

## Seismic Ambient Noise Before and After the Lockdown

**Figure 2:**
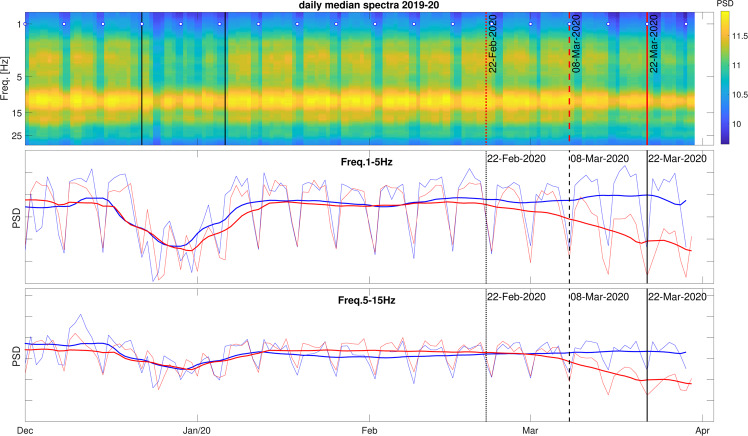
Spectrogram of ambient signal recorded on the vertical component of station MILN, December 1^*st*^ 2019-present (top); the same spectrogram, averaged (red lines) at frequencies from 1 to 5 Hz (middle) and from 10 to 15 Hz (bottom), compared with results obtained in the same way, from recordings made December 2018-April 2019 at the same station (blue). (The East-West and North-South components show very similar trends, albeit slightly less pronounced.) The 2018/2019 curve is slightly offset, in order for weekends to be “in phase” with those of 2019/20. To emphasize relatively-long term (weekly rather than daily) effects, curves are also smoothed over time (thicker lines). The dates when specific containment measures were first implemented are marked by a dotted line (lockdowns of eleven municipalities in the North), a dashed line (restriction of movement and closure of schools in the entire country) and a solid line (interruption of all non-essential industrial activities).

The seismic signature of the containment measures in Italy is apparent from a relatively simple analysis of continuous recordings at station MILN, located within the city limits of Milano, in a busy area near the University of Milano campus and the Lambrate train station. We compute spectrograms (Fig. 2) by Fourier-transforming 1-hour-long segments of continuous signal, with a 30-minute overlap between subsequent segments; for each calendar day, all segments are then averaged, and the squared modulus of the resulting average Fourier transform is computed: this way, a single “power-spectral density” (PSD) function is obtained, for each station, component (East-West, North-South, vertical) and calendar day.

Figure 2 shows clearly that the lockdown has a relevant impact on recorded seismic noise over a broad frequency range; its effect disappears at frequencies below 1 Hz, where anthropic noise is weaker. The energy drop associated with the lockdown is comparable with that occurring every weekend and during the winter break, both in 2018/19 and 2019/20. Interestingly, loss of energy is gradual over time, starting with the first lockdown measures on February 21, and increasing with time until a plateau is reached around March 22 (interruption of non-essential industrial activities). A trend similar to that seen in Fig. 2 has also been found through the analysis of cell phone displacements^18^. This suggests that vehicle traffic, which was significantly reduced (particularly in and around Milano) already with the February measures, contributes significantly to the entire spectrum of anthropic noise; there is also episodical evidence from the press that a number of factories were closed based on the unilateral decision of their owners, before the government-imposed lockdown.

**Figure 3:**
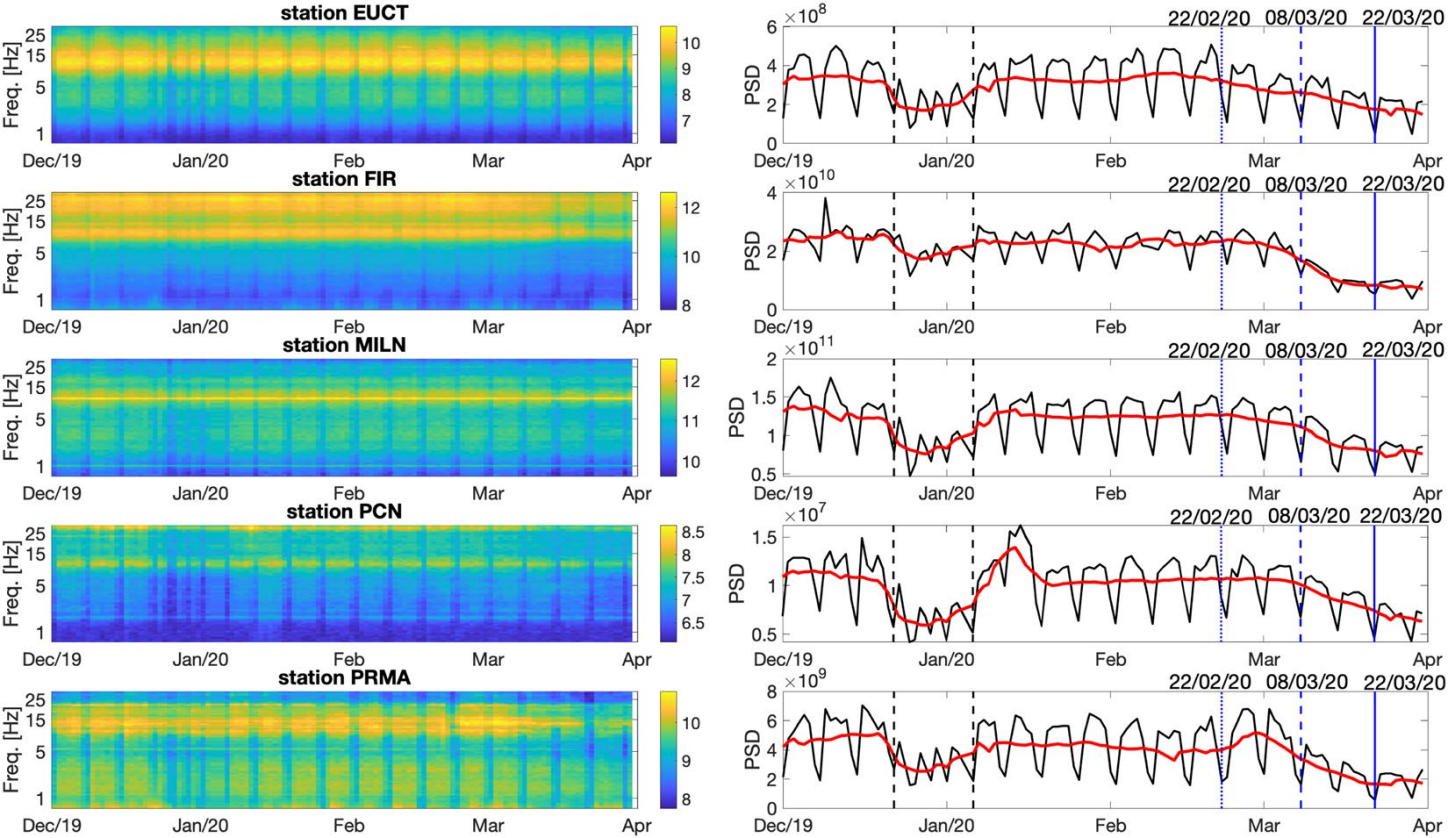
Spectrograms (left) of signal recorded on the vertical components of stations (top to bottom) EUCT, FIR, MILN, PCN, PRMA. To the right, curves obtained from the same spectra, by averaging over the entire frequency range of interest (1 to 40 Hz), without (black line) and with (red) smoothing, similar to Fig. 2. The dates of mentioned governmental decrees are highlighted as in Fig. 2, and so are the typical initial and final days of winter break.

**Figure 4:**
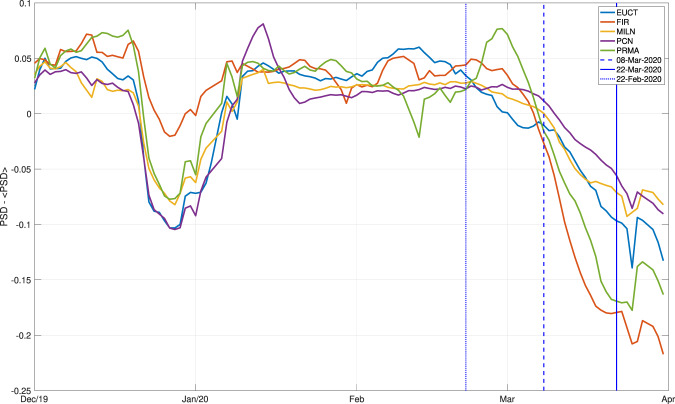
The smoothed red curves shown in Fig. 3 are plotted here on a single graph, for comparison; for each station, the average value of the PSD observed in the time interval of interest is subtracted from the corresponding curve, prior to plotting, as this can change significantly from station to station, but is not relevant to our analysis. Each colour corresponds to one station, as specified. Again, the dates of mentioned governmental decrees are highlighted as in Fig. 2.

The analysis applied to station MILN is repeated for all seismic stations of Fig. 1, and the results are illustrated in Figs. 3 and 4.

Lockdown measures apparently impact all stations under consideration, but the character of their effects changes in various ways with station location. In the case of FIR, located in the city of Florence, the signature of the winter break is almost negligible, while the February/March lockdown still has a prominent effect; it might be possible to interpret this observation through the analysis of anthropic activities usually taking place in the area (e.g., tourism, which is presumably not reduced by the holiday). The drop is gradual at all stations, with no specific governmental decree standing out with respect to the others. At station PRMA, a slight increase in ambient noise occurs after February 21 and before March 9.

## The Spectral Signature of “Cultural” Seismic Noise

**Figure 5:**
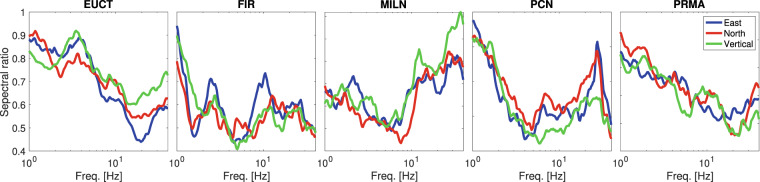
Ratio of PSD after vs. before the lockdown, at stations (left to right) EUCT, FIR, MILN, PCN and PRMA, in the frequency range 1–40 Hz. Different-colour curves indicate different instrument components, i.e. blue, red and green for the East-West, North-South and vertical components, respectively.

We next characterise anthropic noise by evaluating variations in the spectra of seismic ambient noise before and after the implementation of lockdown measures. We compute the ratios of the PSD measured (as described in sec. 3) on Tuesday March 31 2020, to that measured at the same station on Tuesday December 3 2019. We carry out this calculation separately for each component, and for all stations analysed thus far; the results of this exercise are shown in Fig. 5. The energy associated with ambient signal is clearly reduced for all stations, at all frequencies in the range of interest. At each station, PSD ratios change with frequency almost exactly in the same way for all components. Near 1 Hz, all stations show a more or less rapid decline in the PSD ratio, with ambient noise being more effectively reduced as frequency grows. This trend continues all the way to 20 Hz at stations EUCT and PRMA, while other stations show a more complex behaviour. Above 10 Hz station MILN stands out, its PSD ratio growing quickly with frequency.

Anthropic noise is known to be relevant at frequencies above 1 Hz, and to consist of a range of different excitation mechanisms^7,15,19,20^. Natural sources such as rain, wind^21^ and sea/ocean waves are typically characterized by frequencies below 1 Hz, and are obviously not affected by the lockdown. We infer that, by taking the ratio of noise spectra before and after the lockdown, an estimate of the spectral character of anthropic noise is obtained, and the spectra in Fig. 5 can help us estimate the nature of anthropic noise in the region of interest, independent of the lockdown; the frequencies where the PSD of ambient signal is most reduced by the lockdown are those where, in normal times, the contribution of anthropic activities to seismic ambient noise is most important. The fact that most energy loss associated with the lockdown is at frequencies between 1–10 Hz is coherent with what is known of the typical signature of industrial activity and vehicle traffic^22^. Seismic data recorded during the lockdown might be particularly useful in identifying sources of anthropic noise, which could be employed by geophysicists, after the lockdown, e.g. to characterise the upper subsoil by cross correlation of ambient signal^23,24^.

**Figure 6:**
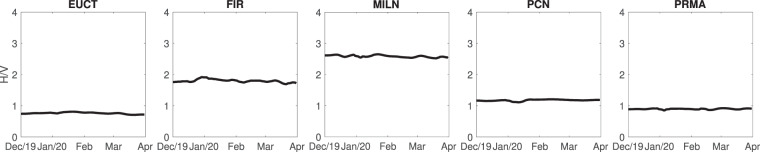
Ratio of horizontal to vertical PSD, averaged over frequency, shown as a function of time from the beginning of December 2019 to the end of March 2020. From left to right, ratios obtained at stations EUCT, FIR, MILN, PCN, PRMA are shown, respectively.

We further analysed the relationship between ambient noise recorded on different components, finding the “*H*/*V*” ratio between the PSDs of horizontal-component and vertical-component signals: first, the PSD of each component of signal recorded on a given day is averaged in the frequency range 1–10 Hz; then the arithmetical average of the resulting East-West-component and North-South-component values is taken; finally, the ratio of the resulting horizontal PSD to the vertical one is computed. The procedure is iterated for each station and for each day between December 1 and March 31, and the results are shown in Fig. 6.

In general, the value of *H*/*V* is related to how seismic energy in the ambient-noise field is distributed in the form of compressional, shear and surface waves^7,25,26^; changes in *H*/*V* after vs. before the lockdown would reveal whether the reduction in anthropic noise affects one of these seismic phases/components more or less importantly than the others; in other words, whether traffic and industry-induced vibration can be associated to one particular constituent of the seismic field. Figure 6 shows that the lockdown measures have no effect on *H*/*V*, and we infer that, while anthropic noise is reduced significantly by the lockdown (Fig. 5), the relative contributions of compressional, shear and surface waves remain approximately constant: the noise wave field is stable in the 1–10 Hz frequency band, and can thus represent a reliable source of information about geological features of the subsoil^7^ or to monitor the evolution of subsurface velocity^10,26^.

## Geographic Distribution of Anthropic Noise

**Figure 7:**
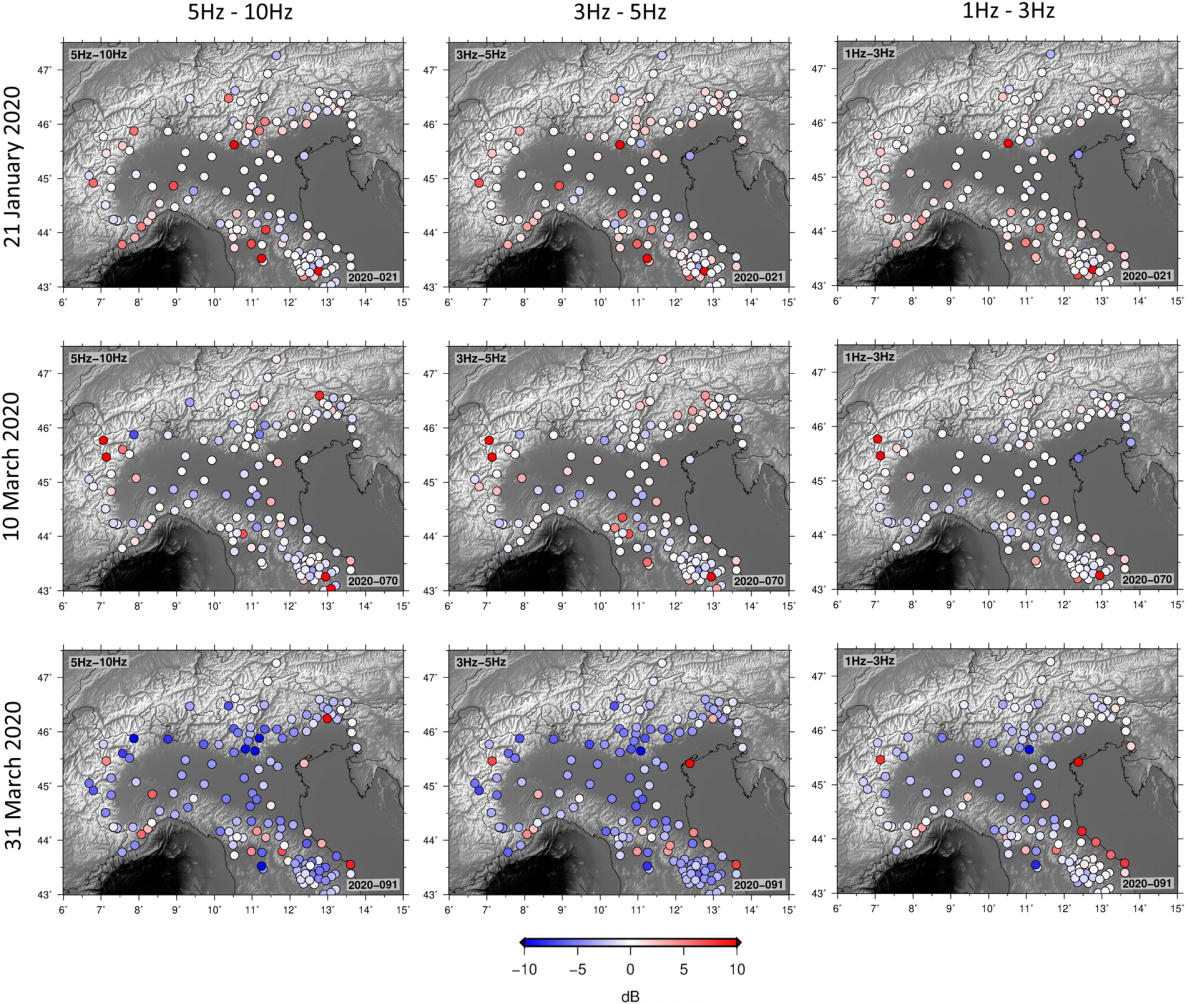
Difference between PSD (6 AM–8 PM) on a given day (top to bottom: January 21, March 10 and March 31: all are Tuesdays), and average PSD calculated for all week days (6 AM–8 PM) from 1–10–2019 to 1-4-2020. The calculation is conducted within three frequency bands (left to right: 5–10 Hz, 3–5 Hz and 3–1 Hz). Values for each station are plotted, through the shown colour code, at the station location.

The Italian territory is densely covered by seismic instruments, and by repeating our analysis on the entire network of available stations we are able to quantify the spatial dependence of anthropic noise reduction. For each station, for each day, the PSD of signal recorded 6 AM to 8 PM is computed, and averaged over different frequency bands. In practice, we employ the direct Fourier method^27^, as implemented in the Obspy package^28,29^: this is standard procedure to identify artefacts related to station operation, episodic cultural noise, overall station quality and level of Earth noise at each site. To emphasize the change in ambient noise with the lockdown, we plot the difference between the values so obtained on three dates in 2020, and reference values obtained conducting the same calculation on data recorded for five months until the lockdown, and averaging. We include as Supplementary Material S1 an animated version of Fig. 7, showing the PSD at the same stations, October 7, 2019 through April 1, 2020; through this time-dependent visualization, the drastic effects of the lockdown are further emphasized.

Our main result, that noise be strongly reduced after the lockdown in the “cultural” frequency range, is confirmed by Fig. 7, and extended to most of Northern Italy. Between 1–3 Hz, the lockdown effects are more pronounced in the Lombardy and Veneto regions than in Central Italy and along the Apennine range. The most important reductions in ambient noise are recorded by stations along the Alpine arc, near Torino, Milano and Verona, and in the city of Florence.

## Summary

We have analysed continuous data from northern Italy, and quantified the effects of the March 2020 coronavirus lockdown on the seismic ambient noise field. We confirm that this effect is significant, and easily observed in our data: see in particular Figs. 3 and 4. The Italian government first imposed a reduction of people (and therefore vehicle) movement, on March 9; we find that this date marks the beginning of a gradual loss in ambient-noise energy at all frequencies, which we attribute to the reduction of road and railroad traffic in the region of interest. Depending on the station, the energy curve flattens out, or starts to decline more slowly towards the beginning of April, despite the more stringent measures imposed at that time (interruption of all non-essential industrial activities). A similar trend has been found from cell-phone displacement data^18^. One implication of our observations is that seismic data could be useful for governmental institutions to monitor the effectiveness of measures involving a reduction or interruption of human activity in a given area.

It is understood that the lockdown only reduces noise of anthropic origin; it follows that by comparing the Fourier spectrum of seismic ambient noise before and after the lockdown (Fig. 5), one can attempt to characterise anthropic noise. We find that, confirming earlier estimates^30,31^, anthropic noise becomes dominant at frequencies above ~2 Hz, where most stations show a ~50% reduction in the energy associated with ambient signal. It then grows with increasing frequency, up to about 20 Hz for most stations. Other than that, the spectrum associated with each station has a unique nature, and knowledge of anthropic activity occurring in its vicinity is probably needed for its interpretation. Our results suggest that seismic data recorded during the lockdown will be useful to identify and characterise specific sources of anthropic noise, which in the future could become useful in local subsoil mapping and monitoring studies^32–37^.

## Supplementary information

Supplementary Movie.

Supplementary Material S1.
