# Supplementary material for: The 2020 coronavirus lockdown and seismic monitoring of anthropic activities in Northern Italy
Source: Sci Rep. 2020 Jun 10;10:9404. doi: 10.1038/s41598-020-66368-0 (PMC7287089; doi:10.1038/s41598-020-66368-0)
Supplement: Supplementary file 2 — Supplementary Material S1. [file 41598_2020_66368_MOESM2_ESM.pdf]

The 2020 coronavirus lockdown and seismic monitoring of anthropic activities in Northern Italy

Evolution of seismic-ambient-noise energy through time, October 7, 2019 through April 20, 2020. Each frame of the movie shows the difference between average PSD for a given week, and a reference average PSD calculated over this entire period of time. The notation and colour code are the same as in Figure 7 of the article, but only one frequency band (3-5Hz) is shown here for simplicity, and, as mentioned data have been smoothed with respect to time by averaging over one week, rather than a single day
